# Communities, health‐care organizations and the contingencies and contradictions of engagement: A case study from Chile

**DOI:** 10.1111/hex.12996

**Published:** 2019-11-12

**Authors:** Cristian R. Montenegro, Nérida Mercado

**Affiliations:** ^1^ School of Nursing Pontifical Catholic University of Chile Santiago Chile; ^2^ Millennium Institute for Research in Depression and Personality (MIDAP) Santiago Chile; ^3^ Independent

**Keywords:** Chile, community participation, community‐institutional relations, decision making, mental health services, organizational, self‐help groups, service‐user involvement

## Abstract

**Context:**

Despite a growing interest in service‐user involvement in mental health services, the interaction between health institutions and local groups is only beginning to receive attention, particularly in global south settings.

**Objective:**

Looking at a participatory initiative in Chile, this study explores how, under unfavourable administrative conditions, health organizations approach and work with communities.

**Methods:**

We interviewed policy‐makers (5), local professionals (10), service users and community representatives (6) linked to a concrete participatory initiative. Participant observation in relevant meetings helped to enrich the interpretations. Thematic analysis was applied to interview transcripts and field notes.

**Findings:**

The findings present a sequence of actions starting with the creation of a network of community‐based groups. A set of problems ensued, related to the group's diversity, internal representation, decision‐making and funding processes. In response, processionals implemented simultaneously bureaucratic and democratic adjustments, developing a vision of community that ignored the particularities—including the motivations—of local groups.

**Discussion and conclusion:**

Based on these findings, we argue that participatory initiatives should be studied as on‐going achievements shaped by broad policy orientations and local configurations of interest. In the process, they produce ad hoc forms of knowledge and visions of community that provide orientation to the agents involved.

## 1 INTRODUCTION

Over the last decades, calls for service‐user involvement have gained prominence in the mental health field. Originally demanded by users themselves, involvement is increasingly framed as a basic aspect in the definition of an adequate mental health system worldwide.^1^

Beyond cursory mentions in policy documents since the 1990s,^2^, ^3^, ^4^in Chile there are no formal mechanisms or guidelines around service‐user involvement in mental health services. Funding for mental health is below WHO standards,^5^ and while recent reforms have secured treatment for specific conditions,^6^ community‐based initiatives are excluded. Unsurprisingly, a recent assessment has identified a ‘low presence and an insufficient degree of organization of service‐user and family groups’.^5^

In another paper^51^, the first author has linked the ‘low presence’ of service‐user groups to policy shifts within and beyond the mental health field. Here, we pay closer attention to how, without guidelines, and facing precarious financial and administrative conditions, local bureaucracies engage with local groups and communities, and how this engagement is sustained over time. We examine the community mental health network (CMHN), a participatory initiative developed in one of the poorest and most populated areas of Santiago. The paper draws on 21 semi‐structured interviews with professionals, users and other community members participating in the CMHN. Thematic analysis was applied to the transcriptions, and the interpretations were enriched by field notes based on seven meetings involving professionals and service‐user representatives.

Participation in health is a polysemic concept involving different practices, goals and rationales.^7^ Most of the literature on participation has been produced in high‐income countries, and it is based on formal projects with clear boundaries in time and space.^8^, ^9^ It usually embraces an evaluative or critical focus, identifying gaps between design and implementation^10^, ^11^ and/or highlighting the political, economic or ideological drives behind participatory discourses and actions.^12^, ^13^, ^14^ With exceptions,^15^, ^16^ the concrete administrative complexity of participatory practices is not documented, especially in low‐resource settings where these initiatives are especially fragile.

The following section briefly characterizes the mental health policy situation and the status of participation in Chile.

### 1.1 Mental health policy and community participation in Chile

‘Community participation's’ relevance for public health in Latin America can be traced back to international and regional processes in the 1970s and 1980s.^17^ Two main antecedents are presented: the ‘community mental health model’ developed in the context of deinstitutionalization (1990‐2005) and the complex effects of recent health reforms (2005 onwards).

#### 1.1.1 From asylum to community

For most of Chilean history, the asylum constituted the dominant response to those deemed ‘mad’.^18^ Community‐based alternatives were explored, following reform process in Trieste and the ‘Movement of Community Psychiatry’ in the United States.^19^, ^20^

Dictatorship blocked these experiments and, between 1973 and 1989, psychiatric hospitals regained their dominance.^21^ In 1980, a neoliberal constitution opened the health system to market forces, while maintaining an underfunded public sector for the poor.

In 1990, coinciding with the transition to democracy, the ‘Caracas Declaration’ was signed by all ministers of health of the Americas.^22^ The Declaration called for the transition from psychiatric institutions to community‐based alternatives.^23^ Although the implementation of these principles has been slow, studies have demonstrated a sustained shift towards outpatient and community‐based services.^24^ This and subsequent plans consolidated the ‘community model of mental health’, a lasting policy framework in the country.^25^

#### 1.1.2 New reforms

At the end of the century, the ‘Universal Access with Explicit Guarantees’ reform was rolled out (AUGE). Becoming effective in 2005, its aim was to expand care to all Chilean citizens suffering from a number of prioritized diseases,^26^ initially including three mental health conditions.^6^

Health care was organized on the basis of individualized diagnosis, treatment packages and costs, without space for community‐led interventions or initiatives. A new National Mental Health Plan (2017) acknowledges the disjuncture between this rationality and the ideas of the community mental health model that ‘limits the effective participation of the community in local health actions’.^4^ ^(p2) Our emphasis^ This contradiction sets the scene for the development of contemporary participatory initiatives.

### 1.2 Approaching participation

Alongside the notion of ‘community’^52^, participation in health is a notoriously diverse concept^27^ that covers notions such as involvement, consultation, public engagement and co‐production.^28^ This diversity accompanies a general lack of conceptual development in the field.^27^

Approaches to participation are usually evaluative, assessing the impact of specific initiatives,^10^, ^29^ or critical, examining the underlying agendas and the broader socio‐political transformations that shape such initiatives.^14^, ^30^ They usually assess formalized participatory projects within clear temporal and/or spatial boundaries, exploring participants’ views and focussing on the dissonance between policy ambition and implementation. In Chile, while notions of community and participation pervade policy discourse for decades, there are no formal participatory mechanisms targeted to mental health service users. Scenarios of informality create tentative and highly contradictory participatory practices, whose emergent characteristics can be rendered invisible through an evaluative or critical approach. How can such initiatives be accounted for? What can be learned from them and in what sense they matter?

For Contandriopoulos,^31^ most approaches to participation in health insist on what participation should be with regard to ideals of democracy and citizenship. A focus on deficiencies neglects the concrete characteristics of participatory projects. Similarly, Cefai et al^32^ call for an approach that assumes the initial in‐determination of participation, the fact that it cannot be deduced from a normative ideal but finds specific forms in different contexts.

David Mosse's ethnography of participatory projects in India attempts to overcome both critical and evaluative approaches, as they ‘divert attention away from the complexity of policy as institutional practice, from the social life of projects, organizations and professionals and the diversity of interests behind policy models and the perspectives of actors themselves’.^33^ ^(p644)^

Although normativity cannot be entirely suspended while studying participation, in this paper we follow these and other authors^34^, ^35^ in an attempt to understand participatory initiatives as localized practical achievements that selectively draw on broader policy orientations and local configurations of interest. Using the CMHN as a case, this paper aims to respond to the following questions: How, in contexts of informality and facing unfavourable conditions, do mental health services attempt to engage and work with community‐based groups, including service‐user initiatives? What do these attempts, in their concrete, tentative expressions, reveal about community participation in mental health more broadly?

## 2 METHODOLOGY

### 2.1 Approach

Given the context‐specific and contingent nature of the initiative, we used an exploratory case study design^36^, ^37^ that provided flexibility to adjust the approach to the emergent characteristics of the situation,^38^ in this case, the CMHN. Twenty semi‐structured interviews and participant observation were conducted between July and December 2015 with a purposive sample of policy‐makers, professionals and service users. Criteria for selection were their participation and/or direct knowledge of to the initiative. This included five respondents from the Mental Health Unit of the Ministry of Health, five professionals working on the mental health team of a health service in the Santiago Metropolitan Region, five professionals working in front‐line mental health facilities and five service users participating in the network.

The interviews were audio‐recorded. Participants were asked to describe the participatory activities that they knew or were involved in and to expand on their views on participation in general, the potential roles of service users and professionals and the broader conditions limiting or allowing participatory actions and community‐oriented work. The interviews lasted between 45 and 120 minutes. Professionals were interviewed in their workplaces. Users were interviewed in primary care facilities, other community‐based spaces or public spaces. A topic guide was used, with questions about participation and specific elements to each type of respondent.

Participant observation was conducted in seven meetings. Four were official CMHN meetings, while the remaining three were aimed at providing local feedback to a National Mental Health Plan draft, with the participation of service users. Participant observation was focused on the service users’ participation in these meetings.

### 2.2 Ethics

The research process was conducted in full accordance with the London School of Economics Research Ethics Policy and Procedure, and formal ethical approval was granted. Interviewees took part under conditions of voluntary informed consent. The nature and aims of the research project were clearly explained both over email and/or phone and immediately before each interview and/or group activity. For the sake of consistency and to reduce possibilities of identification, pseudonyms are used across the paper, and the specific settings are also anonymized.

### 2.3 Analysis

Initially, this research project was aimed at understanding the perspectives of professionals and service users about participation. The topic guide included four broad themes: (a) service‐user roles and contribution; (b) organizational conditions for involvement; (c) institution‐community interactions; and (d) professional roles. Nevertheless, during fieldwork, the existence of on‐going initiatives like the CMHN became apparent. Interviewees talked about the CMHN, their roles and expectations about it, and not about participation as an abstract possibility. The interviews moved from the originally defined topics to an interest in the origins, characteristics and problems of the CMHN.

Thematic analysis^39^ was used to manage the textual material produced by the interviews but, instead of a structure of a‐temporal themes, the views of respondents are anchored to a sequence of situations.^40^Analytically, privileging the temporality of the project serves to highlight its inherent tensions, the problems created and the solutions rehearsed.

### 2.4 Case study setting

The CMHN was developed by a team of professionals working in the mental health department of a health service. A health service (HS) administrates the network of health providers within a group of municipalities, including hospitals, primary care settings and community mental health services. This HS serves the largest population in the country.

According to the interviews and documents collected, in 2005 a local team coordinated an active network of self‐help groups linked to alcoholism. In 2010, the HS used this experience to build its own platform of involvement with community groups across its territory. This was the beginning of the CMHN in its current shape.

This paper examines the context in which this initiative emerged, the tentative schemes of interaction produced and the policy ideas that guided the process, to understand the institutional complexity of participation when translated into concrete strategies, roles and actions.

## 3 FINDINGS

### 3.1 Making contact

In 2005, a local community mental health service (or COSAM) operating in a ‘comuna’ in Greater Santiago developed an active network of self‐help groups linked to problems of alcoholism, domestic violence and depression. In an attempt to standardize the way services worked with local groups, and make the network extensive to its seven constituent comunas, in 2010 the HS used this small‐scale initiative to build a larger platform of involvement called the community mental health network (CMHN), reaching any local group whose actions had an impact on the community's well‐being. The goal was to ‘re‐direct the relation with the community’ (internal document). For Oscar Ulloa, a psychologist that participated in the process, this had two dimensions.

It’s about centralisation in the health service, but it is a decentralisation and expansion across the territories. The original local groups were very homogenous and close in distance. They were mostly self‐help groups linked to pathologies: mostly alcohol, a bit of depression, a bit of domestic violence and that’s it.

The principles contained in the Second National Mental Health Plan^3^ reflect this shift. The plan emphasized prevention, promotion and intersectoral work to address mental health issues. Mental health promotion had to involve a broader set of local partners, marginalizing traditional self‐help groups.

A financial dimension also marked this transformation. Community‐oriented actions had low priority for the mental health system. To fund local self‐help groups, the stable reserves of drunk‐driving fines were used, under the framework of the ‘Law 19.925 on expenditure and consumption of alcoholic beverages’.^41^

Before 2010, these funds were directly transferred to self‐help groups. According to Claudio Farías, administrator of these funds at the central level, this had to change:

Self‐help groups were used to receive an unconditional monthly allowance. We are implementing the idea of a ‘competitive fund’. Each group submits a project. Depending on available funds, the HS decides the amounts and other conditions. (…) groups should use these funds in projects that transcends their membership, expanding their work to the wider community through promotional work.

This new approach to funding was a key element in setting up the CMHN. Before 2010, self‐help groups were funded on the basis of their contribution to either the rehabilitation and support of persons with problematic alcohol abuse (and related issues) or the care of deinstitutionalized psychiatric patients. After the transformation, funding became an *investment*, conditional on the formalization of activities and the development of a project that could make a promotional or preventive impact *beyond* the group. According to Daniela Silva, a social worker (HS) involved in the process.

What we want is to build a movement around mental health (…) we welcomed any group as long as they do promotional and preventive mental health actions.

These changes were also justified by the need to modify the paternalistic relationship between professionals and local self‐help groups that, according to interviewees, characterized the CMHN initial setup. According to Oscar Ulloa:

The original groups had an infantilised view of their needs, they would say ‘no, we are not autonomous’, ‘we can’t do this’. And professionals worked in a very paternalistic way, assuming full responsibility for the groups (…). Professionals never installed capacities and there was no vision of the future.

A focus on promotion above treatment broadened the scope of potential local partners, casting doubts about the relevance of traditional self‐help groups. Funding was conditional to promotional work beyond the groups. These were ways to rationalize the selection of groups, but they also reveal a transformation in how ‘community’ was understood: From being an indifferent space where self‐help groups developed closed activities, ‘community’ became the agent and the target of mental health promotion.

The change was eventually implemented. The original groups were integrated, and new groups were added, attracted by funding. These funds allowed the HS to create new professional roles in each commune, to coordinate the network. Yet, this initial layout created unexpected problems.

### 3.2 The problem of diversity

This change was good, but we need more resources because we’re using money from the Alcohol Law, and this is unfair, because there’s a lot of alcoholism here. (Luz, service user)

Some users said ‘these are alcohol funds, destined to alcohol issues’. What can you say? Of course! We know that, but this is the only source of funding for community‐based work. If the health ministry doesn’t develop another channel, then we’ll have to use these funds. (Miguel, Occupational Therapist, HS)

The alcohol law, aimed at fund alcoholism self‐help groups, was an irregular and fragile funding mechanism and every interviewee agreed on this. However, it was the only source and it gave the HS a mechanism to expand the CMHN. About this, Renata, member of the CMHN and leader of a local initiative helping older people to retain cognitive abilities, said:

The psychologist told me about this new network, so I thought it was important to come to the monthly meetings, because maybe we could get some resources for our projects. That’s why I started to participate, in 2010. We organised ourselves, we submitted two projects and we won. We bought a projector, a printer and a notebook and we had enough to go on a trip with all the grannies.

Like Renata, most newly integrated local groups had previous connections and years of experience obtaining funds from public and private sources. Besides the problematic use of the Alcohol Funds, a new problem emerged: the imbalance between resourceful organizations and other groups that, for many years, need not to compete for funds. Karen Solis, a social worker working directly with the network, expressed:

We made a lot of efforts to develop methodologies to work with them, but these are very different groups. It’s hard to find the thread across them. You have the group of grannies suffering from depression, and they knit. They have no experience in working with other groups, they don’t even read. And you have the Mapuche organisation throwing projects like a machine, with institutional links and a clear political goal. You have the organisation that values everything you do for them and the organisation that finds everything lacking. (…) the challenge was to find the common feeling, the common meaning and the common sense of the meetings. We need to work to understand *ourselves* as a community, as a collective. (Our emphasis)

While the task of centralizing, expanding and funding the network was done, the resulting aggregate of groups seemed to baffle the professional coordinators. Each group had its own background and its own reason to be there, mostly related to funding. Somehow, this was perceived as a limitation. Miguel makes the same point differently:

We sat with this people [the local groups] and asked them ’what should we do here?’, and they said, ‘we are here for the projects’. ‘Ok, but what do you want to do, what is your vision, beyond the projects? Do you want to grow as groups? But why do you want to grow? You want to have more voice? But why do you want to have more voice?’ I mean, we need to aim much higher than just have power for the sake of having power, we need to build something together.

The network’ existence was not promoted or demanded by local groups. Rather, the network was a perfect example of an ‘invited space’^42^ set by an institution for community agents to participate. Even more so, the CMHN deeply transformed an existing way of working with local groups, creating more conditions and forcing them to change. Still, even after accepting the new conditions something was still demanded from the groups: their communion with the network itself.

### 3.3 The problem of leadership and representation

As the initiative moved along, a new regime of engagement was stabilized. This made other problems apparent, closely related to the problem of diversity: the problem of leadership within the groups (also expressed as a problem of representation). As Miguel put it:

Through the meetings with the groups I see a problem of leadership… the lack of participation **within** the organisations. How they share that leadership. You always see the same persons and you ask ‘why doesn’t that lady comes more often?’, ‘Nah, that’s because she doesn’t feel prepared, I can manage everything’ says the leader. But you shouldn’t be managing everything. If you work horizontally, then other representatives can come because, I mean, if only one person comes all the time, then how do we know that her opinion is the opinion of the group? [Our emphasis]

Before 2010, groups received funding and support by virtue of being self‐help groups. Professionals worked *in* the groups. The distribution of leadership and representation within the groups becomes a problem only in the context of the new regime of interaction that, in turn, responds to the process of expansion and centralization initiated in 2010. In the monthly meeting, groups had to be represented by someone and the figures became a negative trait. Rosa, an active service user who led a self‐help group for victims of domestic violence, acknowledges the problem:

So, when you get into this thing called participation, your presence is required. ‘Can you come to this?’, ‘can you stay for this meeting?’… sometimes you don’t have people ready to replace you in every meeting, so you repeat yourself and that’s not ideal.

For her and other service users, representativity requires the permanent effort of being recognized as reliable, knowledgeable and connected. Accepting invitations helps to sustain this recognition, confirms this reliability and provides an opportunity to learn and create connections. It is, within limited resources, the main labour of participation. However, doing all this undermines their ability to distribute their representation in the context of the network. While aimed at fostering autonomy, the new regime of engagement placed the groups’ internal operation as a concern—and an object of intervention—for professionals.

### 3.4 The problem of coordination

The new regime of contact created additional difficulties. The network was large, and the monthly meetings packed with information—from the professionals to the groups—and activities aimed at identifying what the groups had in common. Additional, participation was inconstant. These meant that critical decisions—about funding distribution and other general concerns—could not be made in the meetings. They could not be made by the professionals either—that could damage the legitimacy of the network. The solution was to elect a decision‐making ‘committee’. Laura, psychiatrist and head of the mental health of the HS, commented:

The committee is the representation of the network. Each commune is represented by a user and a professional. ‘But it’s going to be too big!’. Well, it has to be big because big decisions are made about the network. We want the network to feel represented by the committee, we want to legitimate this space because we still have problems. Members of the local groups come here and ask ‘who did you ask about this?’, ‘the representative of your commune came‘, ‘but, we don’t have a representative’, ‘ok but we do this at the beginning of every year!’. And you always have the same problems.

Five members from different community groups, elected by their peers in an annual assembly, composed the committee. Five professionals also participated, with a ‘permanent user member’ breaking the tie. The committee was an important democratic and organizational achievement. It could made binding decisions, reacting quickly to external requirements and opportunities. Still, its legitimacy seemed at risk, as indicated by Laura. This is linked to the problem of diversity. If the network was merely a collection of self‐interested parties, reaching consensus was a complex and slow task. Decisions required a degree of unity across the networks’ members. Besides being a desired characteristic of the network, ‘unity’ was a bureaucratic pre‐condition for legitimate decision making.

### 3.5 The problem of selection

Before 2010, the distribution of funds was arbitrary. We opened the funds and that created resentment because the cake was divided in more slices. But that’s not an issue anymore. The discussion now is ‘what do we fund with this money? Do we fund the ladies that knit or the Mapuche group?’. We don’t have preferences, the point is to do community‐work. So, the committee decided that, starting this year, the funds would be divided in fixed amounts for each commune, and all the groups within a commune would present a single, territorially‐relevant proposal. (Daniela Silva).

Regardless of the democratic and administrative adaptations, the problem of selecting projects—and therefore, groups—to be funded was still there. Decisions remained contested, even after the creation of the committee, so a new adaptation was necessary. Instead of funding individual groups, the total funds were divided proportionally between the seven communes: groups within a commune to work collectively on a single project. According to Cesar Ayala, the permanent user member and originator of this change:

The funds needed a territorial utilisation. This is not going to work at first, we know that, but it is good to acquire a sense of territoriality. This relates with the difference between the herd and the pack. In the herd, everybody does the same and they need a shepherd to protect them. In the pack, you preserve your individuality and work around shared goals. In the herd the shepherd feels like the owner of the group, but in the pack we all own the group.

This decision was congruent with the professionals’ view of autonomy, unity and transcendence: groups of all sizes and orientations had to know each other and work together without the assistance of professionals, to impact their communities. However, simultaneously, this was a way for the committee—and the HS—to disburden itself from the complexities of selection and its inherent tensions.

## 4 DISCUSSION

The findings described the tensions between organizational demands, the origins and development of the network, and the adverse institutional conditions faced. Community engagement was presented as a contingent sequence of problems—and ensuing decisions—, identified and managed by a team within a health‐care organization. Approaching engagement as instances of adjustment and decision reveals its contradictory character in the face of adverse institutional conditions. In this section, we highlight these contradictions and what they reveal about participation and informality, in the light of current literature.

### 4.1 The meaning of community as an object of professional knowledge

The network was born out of an effort to rationalize community engagement, making it extensive to a wider set of groups. This was done through competitive funds, prioritizing autonomous local initiatives working to impact their communities. The funds attracted groups with different interests, agendas and experiences. In managing this diversity, a distinction emerged between the *actual* set of groups and the ‘meaning’ of the network, the unifying thread across the groups.

For professionals, participation meant understanding and embodying this meaning. They *knew* what legitimate, meaningful community‐oriented work was. This knowledge was not based on an explicit definition of participation. It emerged contingently as a way to control and navigate the engagement process and its consequences. This professionalized interpretation of community was applied to issues of leadership, representation and the internal organization of local groups.

The literature on participation has placed a strong focus on the professionalization of community groups.^43^, ^44^, ^45^ The findings from this study, on the other hand, demonstrate how professionals navigate the diversity of local groups through what Vogt calls a logic of reflexion and balance^46^ that allow them to make decisions.

### 4.2 Institutionalization as tentative process

The risks and realities of institutionalization and domination of community groups have been documented in the literature on service‐user involvement.^12^, ^13^, ^14^ This article contributes by showing the graduality of this process. The creation of a decision‐making committee was a way for the initiative to respond more quickly and productively to the pace of the institution. ‘Unity’ among the local groups was valued because it facilitated the work of the committee, funding territories—instead of groups—was another way of reducing the complexity—and potential illegitimacy—of decisions.

In this sense, besides the task of aligning and distinguishing groups according to a professional vision of community and participation, such adjustments adapted participation to the timings and structures of the organization. The diversity, autonomous interest and forms of solidarity of the groups were gradually moulded according to the rules of the organization. This reduced the risk of contestation and immunized the network—and, more importantly, the professionals *in charge* of it—from the potential illegitimacy of their decisions.

### 4.3 Concrete paradoxes and the horizon of community

Nonetheless, each of these bureaucratic decisions was framed as steps towards a more participatory and democratic relationship between the organization and local groups. A vision of community, impact and autonomy guided the initiative from the beginning and continued to operate in front of each problem. In this sense, each decision carried bureaucratic and democratic aspirations. Professionals struggled to translate these contradictions into a role for themselves. Ideas of community emerged, once and again, as a way to make sense of their work, a horizon where their actions—and those of the groups—could and should be oriented, a vision that was bigger that what each member needed or wanted from the initiative. Paradoxically, the concrete and diverse interests of the local groups that the initiative successfully congregated were perceived, by professionals, as a barrier in the path towards such horizon.

## 5 CONCLUSION

On the basis of a case study in Chile, this paper explored the ways in which local health services attempt to work with community groups, under precarious financial and administrative conditions. By approaching participation in its initial in‐determination and as a time‐based, tentative process, we have shown how attempts at engagement lead to unexpected scenarios and problems, and how forms of knowledge and visions of participation and ‘community’ emerged in the process.

Dynamics of professional and/institutional control have been observed and denounced by researchers and users.^12^, ^45^, ^47^, ^48^ As seen in the introduction, co‐optation and control of users’ views and influence is part and parcel of participatory projects.^49^ Attention to domination is an important contribution of the social sciences, and efforts should be made to understand it as a gradual and tentative process that responds to the conditions generated in the course of participatory initiatives. Following Cornish's call for prioritizing the ‘concrete’ in community health research,^50^ we call for more research exploring how, in different settings, participation actually works, what form does it take and what does it *do* to health organizations, local groups and their interaction.

In this way, participatory efforts appear as sequences of actions and reactions that slowly stabilize contingent regimes of interaction, but whose fragility never disappears. Professionals make efforts to introduce a sense of continuity across this contingency, through actions and forms of knowledge^33^ that orient their work with a diverse set of groups.

In terms of policy‐making, considering the growing pressure towards the participation of mental health service users, careful attention should be paid to how local‐scale initiatives develop over time. This not in terms of their efficacy or as ‘best practices’^33^ but as real‐life experiments that reveal the administrative complexity of participation and allow for the anticipation and management of dilemmas. Emphasis should be placed on the reciprocal learning processes characterizing participation, beyond narrow definitions of outcomes or prevalent normative parameters. A focus on local specificity should be privileged over scalable participatory layouts.

## CONFLICT OF INTEREST

Cristian R. Montenegro and Nérida Mercado declare that they have no conflicts of interest.

## Data Availability

Data are available on request from the authors.
